# Exosomal microRNA miR-92a concentration in serum reflects human brown fat activity

**DOI:** 10.1038/ncomms11420

**Published:** 2016-04-27

**Authors:** Yong Chen, Joschka J. Buyel, Mark J. W. Hanssen, Franziska Siegel, Ruping Pan, Jennifer Naumann, Michael Schell, Anouk van der Lans, Christian Schlein, Holger Froehlich, Joerg Heeren, Kirsi A. Virtanen, Wouter van Marken Lichtenbelt, Alexander Pfeifer

**Affiliations:** 1 Institute of Pharmacology and Toxicology, University Hospital Bonn, University of Bonn, Sigmund-Freud Strasse 25, 53127 Bonn, Germany; 2 Research Training Group 1873, University of Bonn, Bonn 53127, Germany; 3 Department of Human Biology & Human Movement Sciences, NUTRIM School for Nutrition and Translational Research in Metabolism, Maastricht University Medical Center, Maastricht 6200 MD, The Netherlands; 4 Department of Biochemistry and Molecular Cell Biology, University Medical Center, Hamburg-Eppendorf 20246, Germany; 5 Bonn-Aachen International Center for Information Technology (B-IT), Institute for Computer Science, University of Bonn, Bonn 53113, Germany; 6 Turku PET Center, Turku University Hospital, Turku 20520, Finland; 7 Turku PET Center, University of Turku, Turku 20520, Finland; 8 PharmaCenter, University of Bonn, Bonn 53127, Germany

## Abstract

Brown adipose tissue (BAT) dissipates energy and its activity correlates with leanness in human adults. ^18^F-fluorodeoxyglucose (^18^F-FDG) positron emission tomography coupled with computer tomography (PET/CT) is still the standard for measuring BAT activity, but exposes subjects to ionizing radiation. To study BAT function in large human cohorts, novel diagnostic tools are needed. Here we show that brown adipocytes release exosomes and that BAT activation increases exosome release. Profiling miRNAs in exosomes released from brown adipocytes, and in exosomes isolated from mouse serum, we show that levels of miRNAs change after BAT activation *in vitro* and *in vivo*. One of these exosomal miRNAs, miR-92a, is also present in human serum exosomes. Importantly, serum concentrations of exosomal miR-92a inversely correlate with human BAT activity measured by ^18^F-FDG PET/CT in two unique and independent cohorts comprising 41 healthy individuals. Thus, exosomal miR-92a represents a potential serum biomarker for BAT activity in mice and humans.

## Abstract (web summary)

Exosomes are RNA-containing lipid vesicles with roles in inter-tissue crosstalk. Here the authors show that exosome release from brown adipocytes is increased upon thermogenic activation, both *in vitro* and *in vivo*, and demonstrate that serum levels of exosomal miR-92 reflect brown fat activity in humans.

## Graphical abstract

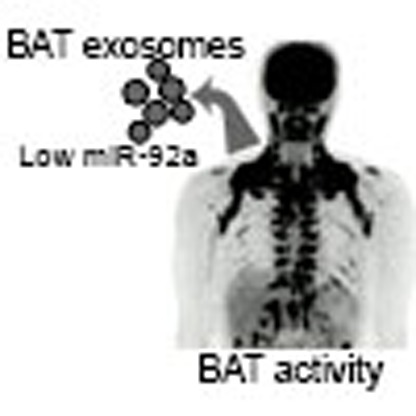

Brown adipose tissue (BAT) is a specialized fat depot that dissipates energy to generate heat^1^,^2^,^3^,^4^. BAT was thought to be active only in rodents and human infants^5^, but recently metabolically active BAT was also found in adult humans^5^. The activity and amount of BAT is usually analysed by ^18^F-FDG PET/CT imaging^6^,^7^,^8^,^9^. However, this technique requires cold activation of BAT and exposes patients to ionizing radiation.

Activation of BAT is mainly controlled by the sympathetic nervous system^1^,^3^. Sympathetic nerves release norepinephrine (NE) and co-transmitters^10^ that activate G protein-coupled receptors and induce production of cyclic AMP (cAMP) which in turn activates protein kinase A and lipolysis^1^.

Apart from ‘classical' brown adipocytes, inducible brown adipocytes with thermogenic potential—also known as beige or brite (brown-like-in-white) adipocytes—have been identified in white adipose tissue (WAT)^3^,^4^,^11^,^12^. ‘Browning' of WAT, that is, increasing the number of beige/brite adipocytes, can be induced by a large spectrum of substances and stimuli including cold-exposure and NE treatment, as well as adenosine^10^ and cyclic GMP^2^. The metabolically active fat depots in the neck and supraclavicular region of human adults contain both constitutive brown and inducible beige adipocytes^13^,^14^,^15^ with deeper neck depots possessing classical brown characteristics and more superficial depots expressing beige markers^16^. Thermogenesis mediated by both brown and beige/brite adipocytes is dependent on the action of the uncoupling protein 1 (UCP-1)^17^, which uncouples mitochondrial ATP production and is stimulated by fatty acids that are liberated by lipolysis^1^. Although the major focus of BAT research has been on pathways that regulate UCP-1-mediated energy expenditure to identify potential BAT-centred therapies, diagnostic tools that allow for safe and easy assessment of BAT in humans are lacking.

miRNAs are small non-coding RNAs, which regulate protein expression in a broad range of tissues^18^,^19^,^20^. miRNAs play an important role in BAT^21^ and several miRNAs have been identified that control brown adipocyte differentiation including miR-106b and miR-93 (ref. 22), miR-133 (ref. 23), miR-378 (ref. 24) and miR155 (ref. 25). miRNAs also regulate browning of white adipocyte and thus the number of mature beige adipocytes^25^,^26^. Interestingly, BAT activation itself, for example, by cold-exposure is able to change miRNA expression patterns of brown adipocytes^23^.

Exosomes are small lipid vesicles 40–100 nm in size^27^ that contain RNAs (mRNA and miRNAs), as well as proteins and surface molecules^28^. Exosome secretion from releasing cell and subsequent uptake of exosomes by a recipient cell provides a mechanism for cell-to-cell communication and exchange of material between cells^29^. The composition of exosomes changes during cellular differentiation and pathological processes^28^. Therefore, exosomes can be involved in pathophysiological processes and can also be used as biomarkers to assess tissue function and disease progression^30^.

To date, there is no biomarker for BAT that can easily be accessed by blood analysis. Here we show that brown adipocytes secrete exosomes and that exosomal miRNAs can be used as a potential serum biomarker for BAT activity in humans and mice.

## Results

### Brown adipocytes release exosomes

**Figure 1:**
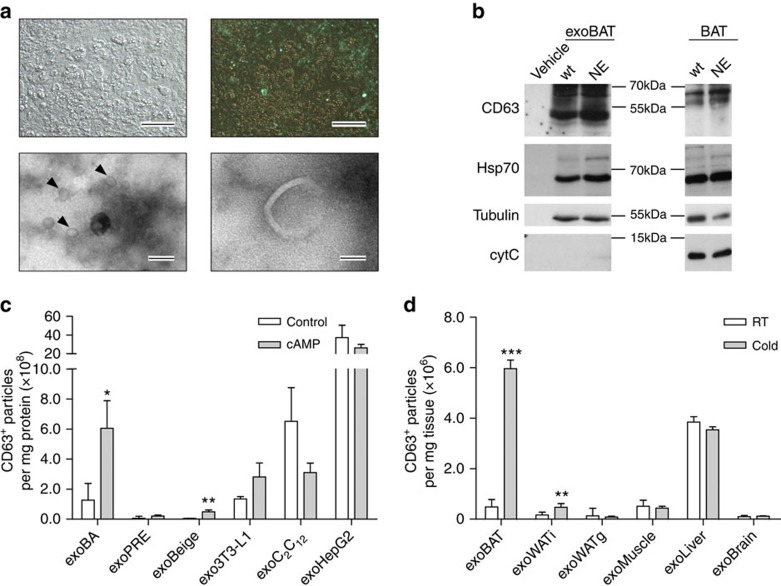
Brown adipocytes secrete exosomes. (**a**) Upper lane: Expression of CD63–GFP fusion protein in murine brown adipocytes. Representative bright field (left) and fluorescence image (right) are shown, (scale bar, 10 μm). Lower lane: electron microscopy images of exosomes in supernatant of brown adipocytes, (left scale bar, 120 nm, right scale bar, 40 nm, arrow heads indicate exosomes). (**b**) Qualitative western blot of exosome marker protein CD63 and Hsp70 expression in exosomes released from BAT (left panel) and in BAT of mice (right panel). The tissue was treated *ex vivo* with 10 μM norepinephrine (NE) or without NE (wt), protein isolation buffer served as vehicle control. Western blotting of tubulin and cytochrome *c* (cytC) are shown as loading control and cellular marker (*n*=1). (**c**,**d**) ELISA quantification of CD63-positive particles released from cells per mg protein before and after cAMP (200 μM) treatment (**c**) and released per mg tissue before and after cold exposure (4 °C for 7 days) (**d**). 3T3-L1, 3T3-L1 differentiated white adipocytes; BA, brown adipocytes; BAT, brown adipose tissue; beige, 12 h 1 μM NE treated or untreated (Control) differentiated white adipocytes; C_2_C_12_, muscle cells; exo, exosomes; HepG2, liver cells; PRE, brown pre-adipocytes; WATg, gonadal white adipose tissue; WATi, inguinal white adipose tissue. Data are presented as mean±s.e.m. (unpaired, two-tailed *t*-test, *P<0.05, **P<0.01, ***P<0.001, *n*=3 for cells, *n*=6 for mice).

To detect exosomes in brown adipocytes, we initially expressed a green fluorescent protein (GFP) fusion protein of the exosome marker CD63 (ref. 31) in murine brown adipocytes (Fig. 1a). Electron microscopy (Fig. 1a) and western blotting revealed the release of exosomes from brown adipocytes and BAT (Fig. 1b). Treatment of brown adipocytes with cAMP, the second messenger that is induced by cold and β-adrenergic stimulation, resulted in a 4.7-fold increase of exosomes in the culture medium (Fig. 1c), indicating that cAMP signalling enhances exosome release from brown adipocytes. To study exosome release also in beige cells, we treated primary murine white adipocytes with NE, which induced a significant increase in UCP-1 expression (Supplementary Fig. 1a). As ‘pure' white adipocytes, we used 3T3-L1 cells that did not significantly increase UCP-1 expression after β-adrenergic stimulation with NE (Supplementary Fig. 1a). cAMP induced a significant increase of exosome release in beige (10.7-fold) adipocytes, but not in 3T3-L1 white adipocytes or in brown pre-adipocytes (Fig. 1c). The treatment with cAMP also did not significantly affect the release of exosomes from muscle cells (C_2_C_12_) or hepatocytes (HepG2) (Fig. 1c). Next we studied the release of exosomes from tissues after cold-exposure. Exosomal markers CD63 and Hsp70 were detectable in all isolations (Supplementary Fig. 1b). Similar to the *in vitro* data, both BAT and inguinal WAT (WATi) of mice showed a significant (9.05-fold and 7.62-fold, respectively) increase in exosome release after cold-exposure (Fig. 1d). WATi was used for the analysis of beige fat, given its high capacity for browning after cold-exposure^32^. In contrast, gonadal WAT (WATg)—the purest WAT depot^33^—and other tissues (that is, muscle, liver and brain) exhibited no significant changes of exosome release after cold-exposure (Fig. 1d). Considering the weight of the three adipose tissues (Supplementary Fig. 1c), 87% of the total amount of exosomes is secreted from BAT under cold conditions, whereas exosomes from WATi and WATg make up only 11% and 2%, respectively (Supplementary Fig. 1d).

### Exosomal miRNAs in serum and release from brown adipocytes

Next, miRNAs were profiled in exosomes isolated from serum of mice with activated BAT—either exposed to cold or treated with the β_3_-adrenoreceptor agonist CL-316,243 (CL)—and compared with control mice, as well as to miRNAs present in exosomes released from brown adipocytes treated with and without cAMP. Based on the expression profiles of the 188 miRNAs that were detected in treated mice and brown adipocytes (Supplementary Table 1), respectively, we computed an average linking hierarchical clustering. The resulting dendrogram (Supplementary Fig. 1e) shows a higher similarity of *in vitro* samples to each other compared with serum samples.

**Figure 2:**
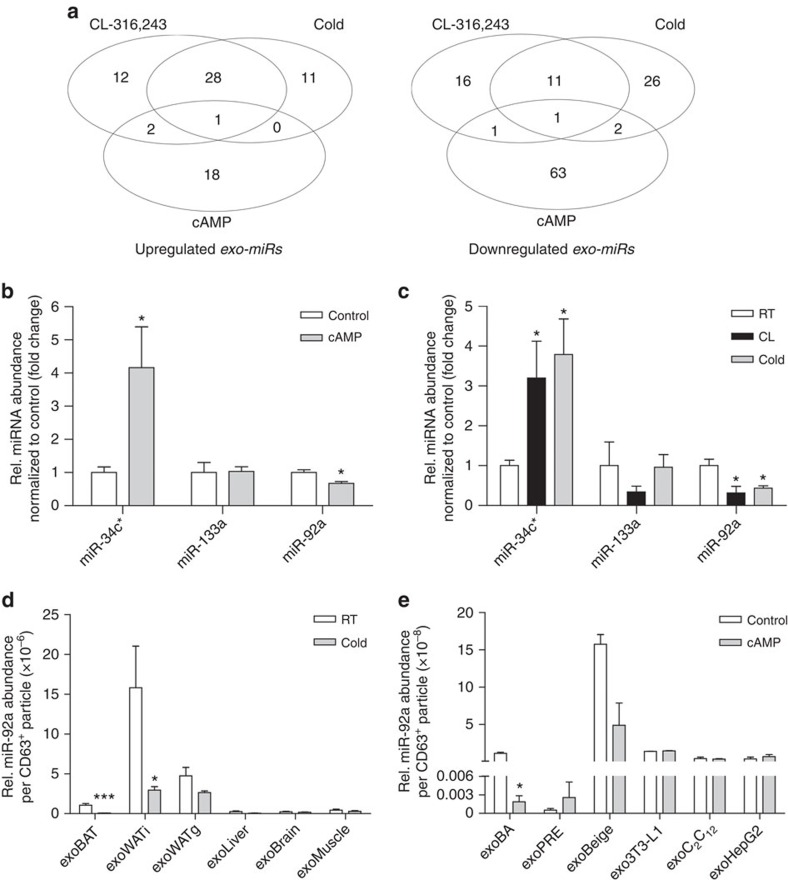
Venn diagram of miRNAs significantly up- or downregulated in the different models. (**a**) Venn diagram showing the overlap of commonly changed miRNAs in mice treated with CL-316,243 or exposed to 4 °C (Cold), as well as in murine brown adipocytes treated with cAMP (200 μM). For further information see also Supplementary Tables 1–3 and Supplementary Figs 1 and 2. (**b**,**c**) qPCR validation of changed miRNAs in exosomes released from brown adipocytes (**b**) and exosomes present in mouse serum (**c**). (**d**) Relative miR-92a abundance per exosome released from tissues after 7-day cold (cold) exposure and RT controls. (**e**) Relative miR-92a abundance per exosome in cells exposed to cAMP versus controls. 3T3-L1, 3T3-L1 differentiated white adipocytes; BA, brown adipocytes; BAT, brown adipose tissue; beige, 12 h 1 μM NE treated or untreated (Control) differentiated white adipocytes; C_2_C_12_, muscle cells; HepG2, liver cells; exo, exosomes; PRE, brown pre-adipocytes; WATg, gonadal white adipose tissue; WATi, inguinal white adipose tissue. Data is normalized to U6 expression and presented as mean±s.e.m. (unpaired, two-tailed *t*-test, *P<0.05, ***P<0.001, *n*=3 for cells, *n*=6 for mice).

To identify miRNAs associated with BAT activation *in vitro* and *in vivo*, we visualized the overlap of miRNA expression between the different groups by Venn diagrams (Fig. 2a). Among 757 profiled murine miRNAs, serum levels of 41 miRNAs were altered by more than twofold in response to CL treatment or cold-exposure (Fig. 2a and Supplementary Table 1). About 12 and 29 miRNAs were up- and downregulated, respectively, in both *in vivo* groups (Fig. 2a and Supplementary Table 1). These miRNA candidates might represent possible biomarkers for BAT activity. Therefore, we analysed next the miRNA profile of exosomes released from brown adipocytes. To induce brown adipocyte activation *in vitro*, we used cAMP, the second messenger that is induced by cold and β-adrenergic stimulation, and compared the *in vivo* with the *in vitro* miRNA pattern (Fig. 2a and Supplementary Table 1). Seven candidates showed the same trend in at least two of the investigated groups; and three out of these seven showed a coherent trend in all three groups, namely miR-92a, miR-133a and miR-34c* (Supplementary Tables 1 and 2).

Quantitative real-time PCR (qPCR) revealed that miR-34c* and miR-92a were significantly up- and downregulated, respectively, in the exosomes from cAMP-treated brown adipocytes and from serum of mice with active BAT (Fig. 2b,c). Although miR-133a was downregulated in serum-derived exosomes of CL-treated mice, miR-133a expression was neither significantly altered in brown adipocyte-derived exosomes nor in any of the mouse models analysed (Fig. 2b,c). Comparison of the abundance of these miRNAs in exosomes and in brown adipocytes revealed that miR-92a and miR-34c* were differentially expressed after cAMP treatment in the exosomes but not in BA (Supplementary Fig. 2a,b). Although miR-34c* is differentially expressed during differentiation of murine brown adipocytes^34^, miR-34c* was not detectable in human serum samples (see below). Therefore, we focused in the current study on miR-92a. Levels of miR-92a in exosomes released from cAMP-stimulated brown adipocytes were reduced after 4 h and a significant reduction was observed after 24 h (Supplementary Fig. 2c). To identify the source of miR-92a in mice, we quantified the amount of miR-92a in exosomes isolated from BAT, WATi, WATg, skeletal muscle, liver and brain. miR-92a was detectable in all tested samples (Fig. 2d). The highest abundance of miR-92a per exosome was released by WATi followed by WATg, BAT, liver brain and muscle (Fig. 2d). Cold-exposure induced a significant downregulation of exosomal miR-92a in BAT (to 6% of the control) and WATi (to 18%), but not in exosomes derived from other tissues (Fig. 2d). Similar changes were observed *in vitro* (Fig. 2e). In mice, the increase in BAT mass on prolonged cold-exposure significantly correlated with the reduction of miR-92a abundance in serum (Supplementary Fig. 2d,e). Moreover, we studied miR-92a release in murine obesity. As expected, mice fed a high fat diet (HFD) for 16 weeks showed ‘whitening' of BAT with a significant increase in lipid droplet size and triglyceride content (Supplementary Fig. 2f,g). Whitening of BAT was accompanied with an increased release of miR-92a from BAT (2.75±0.65-fold) and increased exosomal miR-92a levels in serum (2.66±0.19-fold) (Supplementary Fig. 2h).

### Exosomal miRNAs in human serum

To analyse whether BAT activity correlates with miRNA in circulating human exosomes, we investigated serum exosomal miR-92a levels in two cohorts of subjects that were studied by ^18^F-FDG PET/CT imaging. Initially, we focused on a well-characterized, coherent cohort (cohort 1) of 22 lean individuals with a mean age of 21.5±2.3 years and a mean body mass index (BMI) of 21.3±1.7. These individuals exhibited considerable differences in BAT activity quantified by standard uptake values (SUV) and glucose uptake rate (Table 1). A description of the subject characteristics can be found in Supplementary Tables 3 and 4. To further substantiate our findings, we analysed a second cohort (cohort 2) with 19 individuals (mean age, 36.3±9.6; mean BMI 23.6±2.4) (Table 2 and Supplementary Table 5).

### Exosomal miR-92a in serum reflects brown fat activity

For the analysis of human exosomal miRNAs, we focused on miR-92a and miR-133a, whereas miR-34c* was not detectable in human serum exosomes. Although miR-92a and miR-133a expression levels showed considerable inter-individual variation in cohort 1, their expression was not different between males and females and was not related to any other parameter such as age, or BMI.

**Figure 3:**
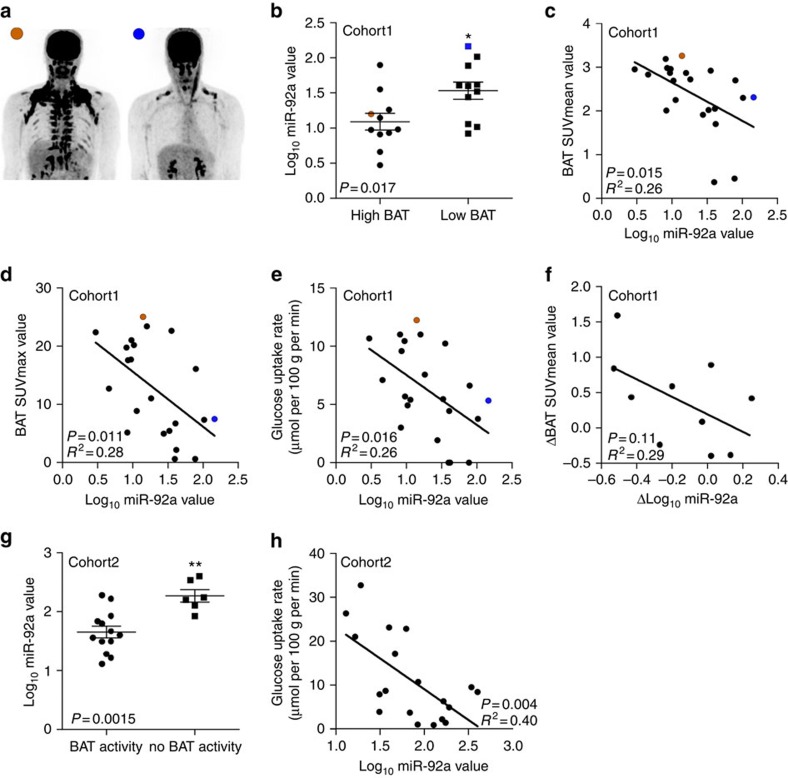
Correlation of thermoneutral exosomal miR-92a in serum with BAT activity measured in humans after acute cold exposure. (**a**) ^18^F-FDG PET/CT image of a subject with high (brown) and low (blue) BAT activity. (**b**) miR-92a expression as quantified by qPCR. Subjects were separated into two groups with high and low BAT activity. Data is normalized to U6, and expressed as mean±s.e.m. (unpaired, two-tailed *t*-test, *P<0.05, *n*=11 per group). (**c**) Log_10_ miR-92a value is negatively related to BAT SUVmean value when considering the whole group. The obtained correlation equation is: BAT SUVmean (predicted)=−0.8749 × Log_10_ miR-92a+3.5227, (Pearson's correlation, *R*^2^=0.26, *P*=0.015, *n*=22). (**d**) Log_10_ miR-92a value is negatively related to BAT SUVmax (Pearson's correlation*, R*^2^=0.28, *P*=0.011, *n*=22). (**e**) Log_10_ miR-92a value is negatively related to BAT glucose uptake rates (Pearson's correlation*, R*^2^=0.26, *P*=0.016, *n*=22). (**f**) Changes in miR-92a expression levels (ΔLog_10_ miR-92a value) tended to correlate with changes in BAT activity (Delta BAT SUVmean value) on a 10-day cold acclimation period (Pearson's correlation*, R*^2^=0.29, *P*=0.11; *n*=10; and Supplementary Table 3). (**g**) Log_10_ miR-92a is elevated in individuals without BAT activity (unpaired, two-tailed *t*-test, **P=0.01, *n*=6 for no detectable BAT and *n*=13 for subjects with detectable BAT). (**h**) Log_10_ miR-92a value is negatively related to BAT glucose uptake rates in a second cohort (Pearson's correlation*, R*^2^=0.40, *P*=0.004, *n*=19).

Based on PET/CT data of cohort 1 (Fig. 3a,b), subjects were initially divided into a ‘low BAT' (mean SUV (SUVmean)<median SUV [2.70]) and ‘high BAT' (SUVmean>median SUV [2.70]) group (*n*=11 per group; Table 1). Interestingly, serum exosomal miR-92a abundance was significantly lower in the high BAT group compared with the low BAT group (unpaired, two-tailed *t*-test, *P*=0.017, Fig. 3b), whereas miR-133a abundance was similar between groups (unpaired, two-tailed *t*-test, *P*=0.757, Supplementary Fig. 3a). In addition, when considering the group as a whole, we observed a significant negative correlation between Log_10_ miR-92a and the BAT SUVmean value (Fig. 3c). Such correlation was absent for miR-133a (Supplementary Fig. 3b). In a stepwise multivariable linear regression analysis with Log_10_ miR-92a as a dependent variable, and age, sex, BMI, fat mass and BAT SUVmean, SUVmax and glucose uptake rate as independent variables, all three BAT parameters were significant predictors of Log_10_ miR-92a (Supplementary Table 6). In addition, individual univariate analyses showed no relations between other parameters and Log_10_ miR-92a. As alternative to BAT SUVmean, we also assessed BAT SUVmax and glucose uptake rate (Fig. 3d,e). In both cases, we observed a significant correlation between these values and the Log_10_ miR-92a value (SUVmax: Pearson's correlation*, R*^2^=0.28, *P*=0.011, glucose uptake rate: Pearson's correlation*, R*^2^=0.26, *P*=0.016, *n*=22), (Fig. 3d,e, respectively). Furthermore, to rule out that single axis logarithmic transformation resulted in such linear correlations, we performed similar analysis for the Log_10_ BAT SUVmean−Log_10_ miR92a (Pearson's correlation*, R*^2^=0.18, *P*<0.05, *n*=22), as well as the Log_10_ BAT SUVmax−Log_10_ miR92a (Pearson's correlation*, R*^2^=0.25, *P*<0.05, *n*=22), which resulted in similar correlations (Supplementary Fig. 3c,d, respectively).

Ten subjects were subsequently exposed to a 10-day cold acclimation period, which caused a recruitment of BAT and an increase in non-shivering thermogenesis, as described previously^35^. Interestingly, miR-92a abundance tended to be lower after this cold acclimation period (19.5±12.9 versus 13.6±12.7) and the change in miR-92a levels tended to be negatively related to changes in BAT activity (Pearson's correlation*, R*^2^=0.29, *P*=0.11, Fig. 3f) on this cold acclimation period. In addition, the regression equation obtained in Fig. 3d correctly predicted whether subjects would either show a large increase (>change in median SUVmean [+0.43]) or a small increase/decrease (< change in median SUVmean) in BAT activity on cold acclimation, based on changes in miR-92a (Supplementary Table 3). The prediction was correct in 8 out of 10 cases with a considerable variation (confidence intervals 0.44 to 0.97 (exact interval)) (Supplementary Table 3). Interestingly, two patients in this cohort did not show BAT activity (BAT SUVmean was 0.37 or 0.45 in these subjects, respectively) along with high serum level of miR-92a (Fig. 3f, Supplementary Tables 3 and 4). One individual participating in the cold acclimation study showed a large decrease in miR-92a after cold-exposure along with high increase in BAT activity (ΔBAT SUVmean=1.59, Supplementary Table 3).

Moreover, we analysed miR-92a levels in serum exosomes in subjects (in 15 out of the 22 subjects blood samples were available; Supplementary Tables 3 and 4) that were acutely exposed to cold (ca. 1–1.5 h). Serum Log_10_ miR-92a levels of these acute cold-exposed subjects also tended to inversely correlate with BAT SUVmean (Pearson's correlation*, R*^2^=0.21, *P*=0.08, *n*=15, Supplementary Fig. 3e). No blood was withdrawn from seven patients due to vasoconstriction in cold (Supplementary Tables 3 and 4).

To verify the observed correlation of exosomal miR-92a with BAT activity, we investigated cohort 2 (19 individuals) with a wider range of BMI (from 18.9 to 28.9) and age (from 21 to 51) that were also analysed by ^18^FDG-PET/CT (Supplementary Table 5). The correlation analysis of Log_10_ miR-92a as a dependent variable, and age, sex, BMI, fat mass and glucose uptake rate as independent variables identified glucose uptake rate as only significant predictor of Log_10_ miR-92a (Supplementary Table 7). Interestingly, exosomal miR-92a abundance was significantly higher in individuals that were BAT negative compared with those with detectable BAT tissue (Fig. 3g). Similar to cohort 1, we observed a correlation between Log_10_ miR-92a and BAT activity (glucose uptake rate) (Pearson's correlation*, R*^2^=0.40, *P*=0.004, *n*=19) (Fig. 3h). Taken together, exosomal miR-92a is an indicator of human BAT activity in the investigated two cohorts.

## Discussion

BAT has received a lot of interest after the discovery of metabolically active adipose depots in the neck and supraclavicular region of adults that take up glucose after cold stimulation^6^,^7^,^8^,^9^. In addition to its metabolic function, BAT is also a source of signalling molecules that can modulate BAT function or even other tissues like the liver^36^,^37^. These BAT-derived signals include nitric oxide^38^,^39^ and adenosine^10^ that activate brown adipocytes in a paracrine/autocrine manner, as well as endocrine factors and hormones like FGF21 (refs 40, 41) and NRG4 (ref. 42). In this study, we demonstrate that brown adipocytes release exosomes that contain miRNAs and that exosome release, as well as the exosomal miRNA pattern change after brown adipocyte activation.

Exosomes are found in various body fluids including blood and play a role in exchanging information between cells and tissues^29^. Several studies have shown that exosomes can regulate the function of the recipient/target cell and miRNAs are considered as major exosomal signals and effectors^29^. For example, exosome-mediated transfer of cancer-secreted miR-105 has been shown to reduce endothelial barrier function; thereby promoting metastasis^43^. Furthermore, tumour-secreted exosomal miRNAs can trigger a pro-metastatic inflammatory response via binding to Toll-like receptors^44^.

The pattern of exosomal miRNAs differ between healthy individuals and patients, as well as in different stages of diseases, therefore, exosomal miRNAs can be used as biomarkers of disease states and tissue function^29^,^45^. The power of exosomal miRNAs as diagnostic tools has been clearly demonstrated for cancer^46^. Moreover, recent studies identified circulating miRNAs as potential biomarkers/indicators of metabolic disorders including obesity (miR-152 (ref. 47), miR-17 (ref. 47), miR-138 (refs 47, 48), miR-15b (ref. 48) and miR-376a (ref. 48)) and type 2 diabetes (miR-593 (ref. 48) and miR-15a (ref. 49), miR-503 (ref. 48), miR-326 and let-7a/f (ref. 50)). Yet, we are the first to relate exosomal miR-92a serum levels with BAT activity in humans.

The miR-17-92 (miR-17, miR-18a, miR-19a, miR-20a, miR-19b-1 and miR-92a) cluster was previously described to be regulated by cell cycle via E2F3 binding and by a negative feedback loop through miR-17 that targets E2F2 (ref. 51). The miR-17-92 cluster is essential for development^52^ and genetic deletion of this cluster results in defects in heart and lung development leading to postnatal death^53^. Dysregulation of several cluster members has been observed in most cancer types^52^ leading to its classification as ‘oncomiR-1'-cluster^54^. miR-92a has been associated with leukaemia, breast cancer and hepatocellular carcinoma^55^,^56^,^57^. Although miR-92 has been shown to impair angiogenesis^58^ and to promote atherosclerosis^59^,^60^, ablation of this miRNA in mice resulted only in bone defects^61^.

While six (miR-17, miR-18a, miR-19a, miR-20a and miR-19b-1) of the seven members of this cluster were investigated in the context of adipocyte differentiation^62^, little is known about the role of miR-92a in adipocytes. First *in vivo* investigations associate genetic knockout and pharmacological inhibition of miR-92a with a HFD-resistant, more healthy phenotype in mice^63^.

The detection of BAT currently relies mainly on PET/CT imaging of ^18^F-FDG uptake into metabolically active BAT. This imaging technique was originally developed—and is still mainly used—for detection of metastasis in oncology. Coincidentally, ^18^F-FDG PET/CT imaging revealed the increased uptake of tracer in a region extending from the anterior neck to the thorax^64^. Several seminal publications^6^,^7^,^8^,^9^,^16^ showed that these areas of cold-induced glucose uptake correspond to BAT. However, the major drawback of this technique is the exposure of patients to ionizing radiation by both the FDG tracer and CT and thus cannot be used for screening purposes or large number of subjects. In addition, this method only visualizes active BAT and consequently has an imminent need to expose subjects to cold.

Thus, diagnostic markers of BAT are of great importance to study the physiological role of human BAT, as well as for clinical trials to stratify subjects and to quantify the effects of drug candidates on BAT. In this regard, the relation between exosomal miR-92a abundance in human blood samples and cold-induced BAT activity is highly promising. However, the correlation is very modest and future studies with larger cohorts are needed, since biomarkers typically need to classify with greater accuracy than an *R*^2^ of 0.40 would allow.

Taken together, miR-92a represents a potential thermoneutral brown/beige fat biomarker for basic science and clinical applications, which can be measured with relative ease in large cohorts of patients.

## Methods

### Study approval

The human studies were approved by the ethics committee of Maastricht University Medical Centre and Ethics Committee of Hospital District of Southwest Finland. All subjects provided written informed consent before any study procedures.

All procedures were conducted according to the principles of the Declaration of Helsinki. All animal experiments were approved by the Animal Welfare Officers of University Medical Center Hamburg-Eppendorf (UKE) and Behörde für Gesundheit und Verbraucherschutz Hamburg and the Landesamt für Natur, Umwelt und Verbraucherschutz Nordrhein-Westfalen.

### Statistics

Values are presented as means±s.e.m. or means±s.d. for subject characteristics. Statistical differences were determined using Student's *t*-test (unpaired, two-tailed). GraphPad Prism 5 or Excel software was used to calculate *P* values; the level of statistical significance was set at *P<0.05. For human serum samples, statistical analyses were performed with SPSS Statistics 20.0 for MAC (IBM, Amonk, NY).

Human serum miR-92a and miR-133a expression levels were not normally distributed in cohort 1 according to Shapiro–Wilk test (*P*=0.000 and *P*=0.001, respectively). Therefore, these data were Log_10_ transformed, which resulted in normal distribution (Shapiro–Wilk test, *P*=0.626 and *P*=0.180, respectively), and analysed accordingly. Two-sided independent sample *t*-tests were used to compare miRNA expressions between groups. Pearson correlations were used to identify correlations between variables.

### Animals

C57BL/6J wild-type mice were purchased from Charles River Laboratories. Cold-exposure was performed by housing 12-week-old male mice at 4 °C for 7 days. The β_3_-adrenergic agonist CL-316,243 (#1499, Tocris, Wiesbaden, GER, 0.2 mg ml^−1^ in 0.9 w/v% NaCl) was administered by s.c. injection (1 μg g^−1^ body weight) for 7 days. Control-treated C57BL/6J mice received vehicle injections (0.9% NaCl) correspondingly. Blood collections were performed after 4 h fasting. Mice were anesthetized with a mix containing ketamin (25 mg ml^−1^), xylazin (0.2%) in 0.9% NaCl and blood was withdrawn transcardially.

For the HFD study, C57BL/6J male mice were purchased from Charles River Laboratories and fed either a HFD (EF acc. D12492 (II) mod., ssniff, Soest, GER) or a normal chow diet (R/M-H; maintenance feed catalogue page 14, ssniff) for 16 weeks starting at the age of 8 weeks. For triglyceride measurements, tissue was weighed and transferred to TGx lysis buffer (150 mM NaCl, 0.05% Triton X-100, 10 mM Tris-HCl and 4% cOmplete (11873580001, Roche, Basel, SUI)). Tissue was chopped with scissors and grinded manually with a pestle. Triglyceride reagent (T2449-10ML, Sigma Aldrich, St Louis, MO) was added according to the manufacturer's instructions. The increase in absorbance at 540 nm is directly proportional to the free glycerol concentration of the sample. The calculated TG content was normalized to the total BAT weight.

### miRNA profiling

qPCRs of exosome-derived miRNAs were performed with TaqMan Rodent MicroRNA Array card A and card B (#4444909, Life Technologies, Carlsbad, CA). Mouse serum samples were pooled (three mice per group) and two pools were analysed per condition (cold, CL or wild type); for the analysis of brown adipocyte-derived exosomes, the supernatant of six wells from a six-well plate was pooled from cells treated with 200μM 8-Bromoadenosine 3′,5′-cAMP (cAMP) and untreated control cells. Data were analysed with software RQ manager1.2.1 and DataAssistv3.01 (Life Technologies).

Exosomal miRNAs that were deregulated more than twofold (compared with vehicle-injected mice) after CL-316,243-treatment or cold-exposure, as well as those miRNAs up- or downregulated twofold after cAMP treatment (as compared with control cells) were considered for the Venn diagram analyses. Selected candidates were validated with TaqMan miRNA assay kit according to the manufacturer's instruction. MicroRNA Assay miR-92 (cat no. 000430, Life Technologies) was used to quantify miR-92a located on chromosome 14: 115044427-115044506 [+] with the following sequence: 3′- UAUUGCACUUGUCCCGGCCUG -5′. U6 snRNA (TaqMan MicroRNA Assays U6 snRNA (cat no. 001973, Life Technologies)) with the following sequence:

3′- GTGCTCGCTTCGGCAGCACATATACTAAAATTGGAACGATACAGAGAAGATTAGCATGGCCCCTGCGCAAGGATGACACGCAAATTCGTGAAGCGTTCCATATTTT -5′ was used for normalization. MicroRNA Assay miR-34c* (cat no. 002584, Life Technologies) was used to quantify miR-34c* located on chromosome 9: 51103034-51103110 [−] with the sequence: 3′- AAUCACUAACCACACAGCCAGG -5′. MicroRNA Assay miR-133a-3p (cat no. 478511, Life Technologies) was used to quantify miR-133a located on chromosome 18: 19405659-19405746 [−] with the sequence 3′- UUUGGUCCCCUUCAACCAGCUG -5′.

### Cell culture

Interscapular brown pre-adipocytes were isolated from newborn C57BL/6J mouse pups (both sexes) and differentiated *in vitro* with hormonal cocktail as previously described^25^. In detail: 80,000 cells were seeded per well on a 6-well plate and cultivated in growth medium (DMEM without pyruvate, 10% FBS, 1% P/S) until 90% confluent. The cells were pretreated with differentiation medium for 2 days containing DMEM with pyruvate 10% FBS, 1% P/S, T3 1 nM, insulin 20 nM followed by induction of differentiation for 2 days with DMEM with pyruvate 10% FBS, 1% P/S, T3 1 nM, insulin 20 nM, 500 μM IBMX and 1 μM dexamethasone. The cells were kept in differentiation medium for another 6 days until fully differentiated. Isolation and cultivation of primary white adipocytes was performed as described^25^. In short: 200,000 primary cells were seeded per well of a 6-well plate and grown in growth medium (DMEM with pyruvate, 10% FBS, 1% P/S) until fully confluent. The cells were induced for 2 days (DMEM with pyruvate, 5% FBS, 1% P/S, 172 nM insulin, 0.25 μM dexamethasone, 0.5 mM IBMX, 1 nM T3, 1 μM rosiglitazone and ABP (50 μg ml^−1^ L-Ascorbat;1 μM d-Biotin; 17 μM Panthothenate)). The cells were kept in differentiation medium (DMEM with pyruvate, 5% FBS, 1% P/S, 172 nM insulin, 1 nM T3 and ABP) for 10 days. After full differentiation, on day 12 browning was induced with 1 μM NE (A7257, Sigma Aldrich) for 12 h and compared with untreated controls. 3T3-L1 (American Type Culture Collection, Rockville, MD) were cultured as previously described^65^. About 200,000 cells were seeded per well of a 6-well plate and grown in growth medium (DMEM with pyruvate, 10% FBS, 1% P/S) until fully confluent. Induction was carried out with DMEM with pyruvate, 10% FBS, 1% P/S, 172 nM insulin, 500 μM IBMX and 1 μM dexamethasone. The cells were maintained in differentiation medium (DMEM with pyruvate, 10% FBS, 1% P/S, 172 nM insulin). HepG2 (HB-8065, ATCC, Teddington, UK) were cultured in growth medium (DMEM with pyruvate 10% FBS, 1% P/S) until fully confluent. C_2_C_12_ myocytes (CRL-1772, ATCC) were fully differentiated as follows: 120,000 cells were seeded per well in DMEM with pyruvate 10% FBS, 1% P/S until confluent then cultured for 7 days in DMEM without pyruvate, 2% Horse Serum, 1% P/S and 1% non-essential amino acids solution (NEAA, cat no. 11140-050, Life Technologies). Medium for all cells was changed every second day. All cells were kept in DMEM with 2% Exo-FBS (#FBSHI-250A-1, System Biosciences, Mountain View, CA) 1% P/S for 48 h either with or without 200 μM cAMP to release exosomes. The supernatant was withdrawn and used for exosome isolation.

### Exosome isolation

C57BL/6J wild-type mice were purchased from Charles River Laboratories. Cold exposure was performed by housing 8-week-old male mice at 18 °C for 7 days followed by 4 °C for 7 days. Blood was withdrawn transcardially after 4 h fasting and subsequently sacrificing the mice. Organs (brain, liver, BAT, WATi, WATg and the soleus muscle) were obtained, washed in ice cold PBS and weighed. Tissues were incubated in 2 ml medium (DMEM with 2% Exo-FBS (#FBSHI-250A-1) 1% P/S) and chopped with scissors. The chopped tissue was centrifuged at 1,000*g* for 5 min at room temperature and re-dissolved in medium and incubated for 30 min at 37 °C, 5% CO_2_. After another medium exchange, the tissues were incubated for 2 h at 37 °C, 5% CO_2_ to release exosomes. The supernatant was then withdrawn and used for exosome isolation. The exosomal samples used for qPCR and ELISA were adjusted to 1.65 mg and 16.5 mg tissue, respectively, to compare exosome/miR-92a release from various tissues. Isolation of exosome either from BA, BAT or serum was performed using exosome precipitation kit according to the manufacturer's instruction (#4478359 or #4478360, Life Technologies)

Exosomal RNAs were isolated according to the manufacturer's instruction (#4478545, Life Technologies). CD63 quantification was done with an ELISA kit according to the manufacturer's instructions (#EXOEL-CD63A-1, System Biosciences).

### Western blotting

Proteins from exosomes, cells and tissues were extracted with RIPA-lysis buffer (150 mM NaCl, 50 mM Tris-HCl pH 7.5, 1% Nonidet P40, 0.25% Na-deoxycholate, 0.1% SDS) containing complete protease inhibitor cocktail (see above), 1 mM Na_3_VO_4_ and 10 mM NaF. Western blotting was performed with anti-CD63 (1:1,000), anti-Hsp70 (1:1,000) and anti-CytoC (1:1,000) antibodies that were purchased from System Biosciences (#EXOAB-KIT-1-SBI) or Santa Cruz (#sc-7159, Heidelberg, GER). Anti-α-Tubulin (1:1,000) antibody was purchased from Upstate (#DLN-09993, Lake Placid, NY) and served as a loading control. Full immunoblots are displayed in Supplementary Fig. 4a–c.

### Electron microscopy and CD63 tracing

The exosomes were incubated with 3.5% uranyl acetate and visualized with a Philips CM10 electron microscope (Philips, Amsterdam, NL). Primary brown adipocytes were transfected with lentiviral particles overexpressing copGFP with a CD63 fusion to track CD63. The cells were differentiated as described above. The tracer kit was purchased from System Biosciences (pCT-CD63–GFP), and used according to the manufacturer's manual. The construct encodes a fusion protein consisting of an organelle-specific signalling peptide and the copGFP fusion with CD63. The organelle-specific signalling peptide directs the copGFP to the appropriate subcellular location. The GFP protein was visualized by excitation at 482 nm and detection at 502 nm with a Leica DMIRE2 Fluorescent Microscope.

### Subjects

miRNA expression levels were determined in thermoneutral blood samples from 2 independent cohorts; cohort 1 comprises 22 randomly selected young, healthy subjects (Table 1) from 2 previously published studies, in which cold-stimulated BAT activity was measured as described previously^35^,^66^. Briefly, BAT activity from static PET scans was measured as mean and maximal ^18^F-FDG uptake in BAT, expressed as SUV: ^18^F-FDG uptake (kBq ml^−1^) per (injected dose (kBq) per patient weight (g)). The investigated regions were manually outlined, using a threshold of 1.5 SUV and Hounsfield units between −10 and −180 to define BAT^67^. When supraclavicular fat tissue activity did not exceed the threshold of 1.5 SUV, fixed volumes of interest were used to quantify tissue activity^68^. BAT SUVmean represents average glucose uptake in all upper body BAT depots. In addition, glucose uptake rates were calculated from dynamic PET data and evaluated with a Patlak curve fitting and a lumped constant of 1.14 (ref. 35). In total, 22 blood samples were withdrawn at thermoneutrality (Fig. 3b–e) and 15 after mild cold activation for 1–1.5 h (Supplementary Fig. 3e) were used to access exosomal miR-92a levels (Supplementary Tables 3 and 4).

Ten subjects were additionally exposed to a 10-day cold acclimation protocol^35^, after which thermoneutral miRNA serum levels were reassessed (Fig. 3f, Supplementary Table 3).

Cohort 2 consists of 19 subjects^69^,^70^ (Table 2). In short, scanning and data acquisition was carried out dynamically, producing tissue time activity curves in BAT. Further, image-derived input function was derived from aortic arch because especially during cold scans blood samples were difficult to obtain. Gjedde–Patlak model was used in graphical analysis, and fractional uptake of ^18^F-FDG was further multiplied with the plasma glucose concentration (mmol l^−1^) to calculate the glucose uptake rate in BAT. The subjects in cohort 2 were sorted for BAT positivity based either on the biopsy finding or on the comparison of warm and cold PET scans (in subjects who did not consent for biopsy). If there was more than threefold higher uptake in cold than warm, subjects were BAT positive^69^,^70^. For image analysis, the investigated regions were manually and semi-automatically outlined, using double thresholding (both CT Hounsfield units and PET radioactivity are utilized). All subject characteristics are displayed in Supplementary Tables 3–5.

## Additional information

**Accession codes:** MicroRNA expression data have been deposited in GEO under accession code GSE79440.

**How to cite this article:** Chen, Y. *et al*. Exosomal microRNA miR-92a concentration in serum reflects human brown fat activity. *Nat. Commun.* 7:11420 doi: 10.1038/ncomms11420 (2016).

## Supplementary Material

Supplementary Information Supplementary Figures 1-4 and Supplementary Tables 1-7

## Figures and Tables

**Table 1:** Human **s**ubject characteristics and BAT activity of cohort 1.

	**All subjects (*****n*****=22)**	**High BAT (*****n*****=11)**	**Low BAT (*****n*****=11)**
Males/females	10/12	7/4	3/8
Age (years)	21.5±2.3	20.8±2.1	22.1±2.5
Weight (kg)	66.0±8.2	65.5±6.8	66.4±9.8
BMI (kg m^−2^)	21.3±1.7	21.0±1.7	21.5±1.7
Fat (%)	21.9±8.1	18.2±6.7	25.6 ±7.8*
SUVmean	2.38±0.77	2.93±0.17	1.82±0.74*
SUVmax	12.68±8.10	19.03±4.48	6.32±5.37*
Glucose uptake rate (μmol per 100 g per min)	6.20±3.82	9.28±2.16	3.12±2.27*

BAT, brown adipose tissue.

Data are presented for all subjects together and the high BAT and low BAT group separately. Values are expressed as means±s.d. (unpaired, two-tailed *t*-test, *P<0.05, high BAT versus low BAT).

**Table 2:** Human **s**ubject characteristics and BAT activity of cohort 2.

	**All subjects (*****n*****=19)**	**BAT activity (*****n*****=13)**	**No BAT activity (*****n*****=6)**
Males/females	6/13	3/10	3/3
Age (years)	36.15±9.71	34.46±9.61	40.33±10.3
BMI (kg m^−2^)	23.42±2.44	23.47±2.63	23.78±2.14
Glucose uptake rate (μmol per 100 g per min)	11.33±9.44	14.55±9.76	3.90±3.95*

BAT, brown adipose tissue.

Data are presented for all subjects together and the no BAT and BAT activity group separately. Values are expressed as means±s.d. (unpaired, two-tailed *t*-test, *P<0.05, no BAT activity versus BAT activity).
