# Supplementary material for: Exosomal microRNA miR-92a concentration in serum reflects human brown fat activity
Source: Nat Commun. 2016 Apr 27;7:11420. doi: 10.1038/ncomms11420 (PMC4853423; doi:10.1038/ncomms11420)
Supplement: Supplementary Information — Supplementary Figures 1-4 and Supplementary Tables 1-7 [file ncomms11420-s1.pdf]

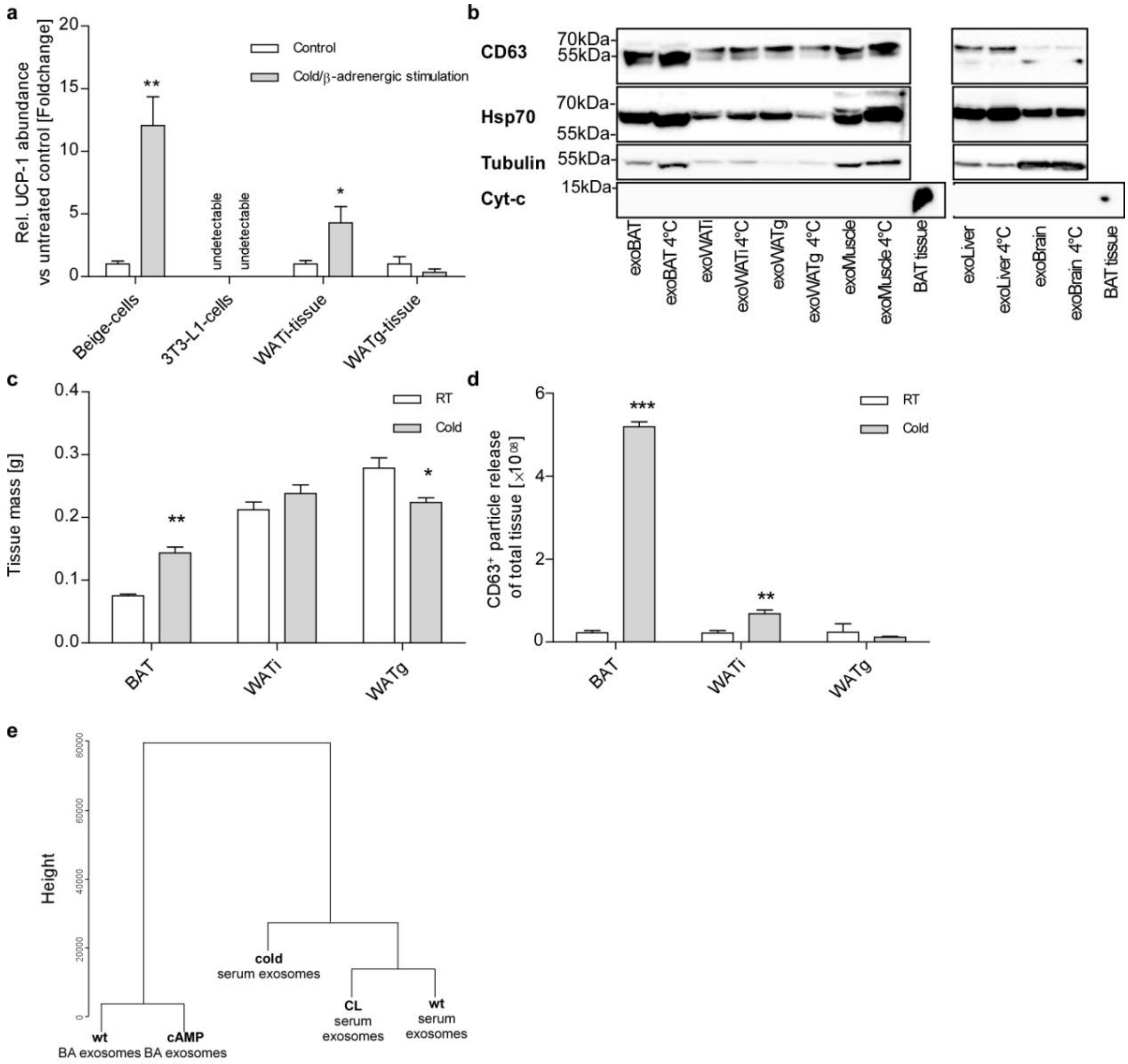

Supplementary Figure 1 Analysis of beige fat and cells and characteristics of exosome release, related to Figure 1

(**a**) Fold-change in UCP-1 mRNA abundance in white adipocytes upon β-adrenergic stimulation (NE) or WAT depots after cold exposure in mice compared to untreated cells or control tissue from mice at room temperature (RT). (**b**) Qualitative Western blot analysis of CD63, Hsp70 and Tubulin positive particles released from tissues and Cyt-c as cellular negative control (*n*=1). The exosomes were released from mice tissues exposed to cold (4°C) compared to RT controls. (**c**)Tissue weights of cold exposed mice and controls (RT). (**d**) Absolute exosome numbers released from fat tissues of cold exposed mice and RT controls (RT). (**e**) Clustering of exosomal miRNAs. Hierarchical clustering of miRNAs in exosomes isolated from serum of mice either exposed to cold (cold) or treated with the β_3 receptor agonist CL-316,243 (CL) compared to vehicle injected control mice (wt), as well as of miRNAs in exosomes released from brown adipocytes (BA) treated with cAMP (cAMP) or untreated

control cells (wt). Values are expressed as mean ± s.e.m. (unpaired, two-tailed *t*-test, $P^{*}\leq 0.05$, $^{**}P\leq 0.01$, $^{***}P\leq 0.001$, $n=6$ mice per group or $n=3$ for cells).

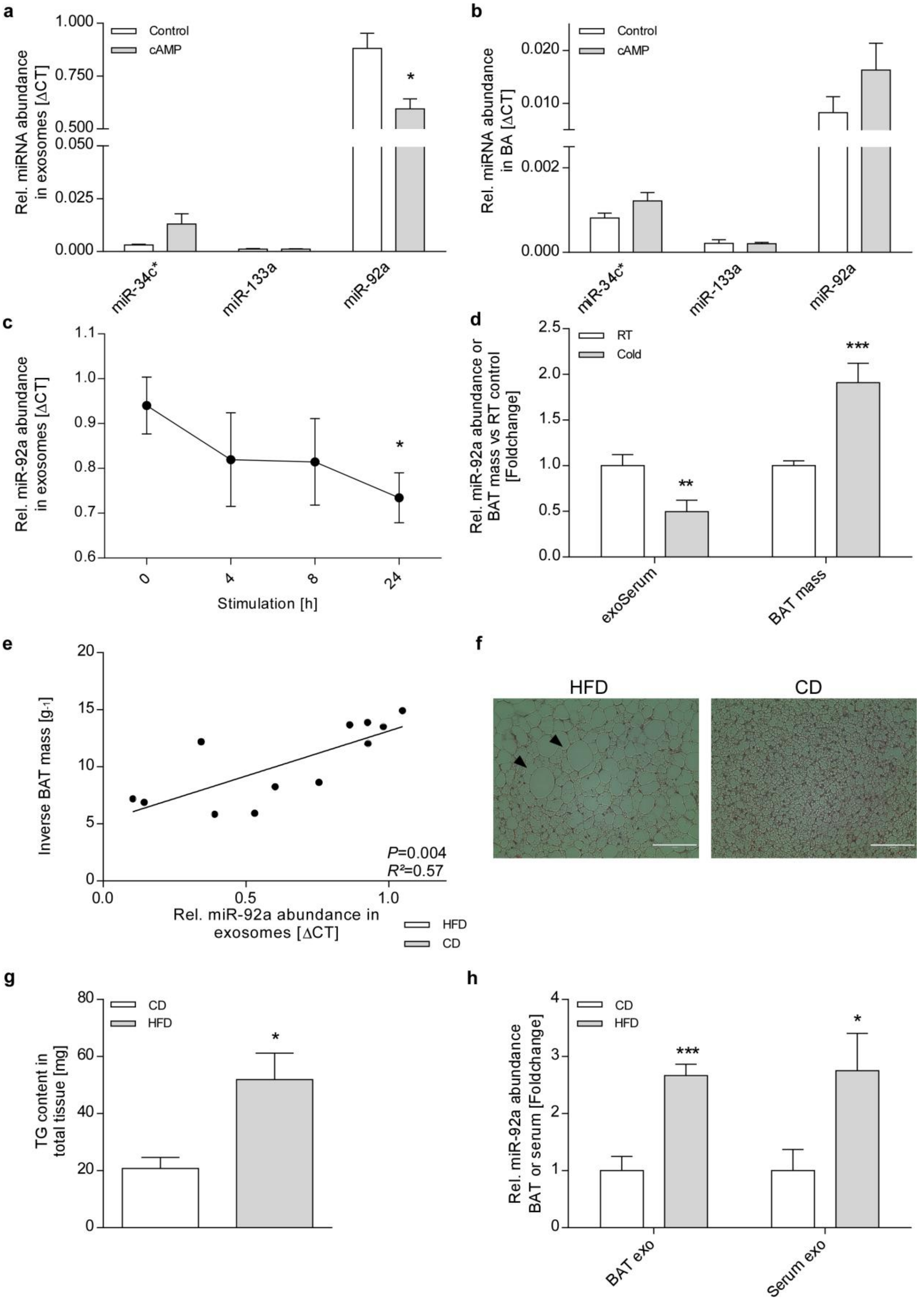

Supplementary Figure 2 Validation of miRNA candidates and HFD study, related to Figure 2

(**a** and **b**) qPCR analysis of the miRNA candidates present in exosomes(**a**) released from untreated (wild type) and cAMP-treated (200 µM) cells, as compared to the cellular miRNA expression (**b**) without and with cAMP treatment (200 µM). (**c**) Time-course of exosomal miR-92a release from brown adipocytes after cAMP stimulation. (**d**) Relative miR-92a abundance in mouse serum and BAT mass compared to cold exposure. (**e**) Inverse correlation of BAT mass with exosomal miR-92a abundance in mice, (Pearson's correlation, R^2=0.57, *P*=0.004, *n*=12). (**f**) H&E Staining of paraffin-sections of 16 week HFD and CD mice, large lipid droplets are indicated by black arrows (Scale bar: 100 µm). (**g**) BAT triglyceride content after 16 week HFD and CD mice (**h**) Total release of exosomal miR-92a from BAT tissues or serum of 16 week HFD mice versus CD mice. Values present mean ± s.e.m. (unpaired, two-tailed *t*-test, **P*≤0.05, ***P*≤0.01, ****P*≤0.001, *n*=5-6 mice per group or *n*=3 for cells).

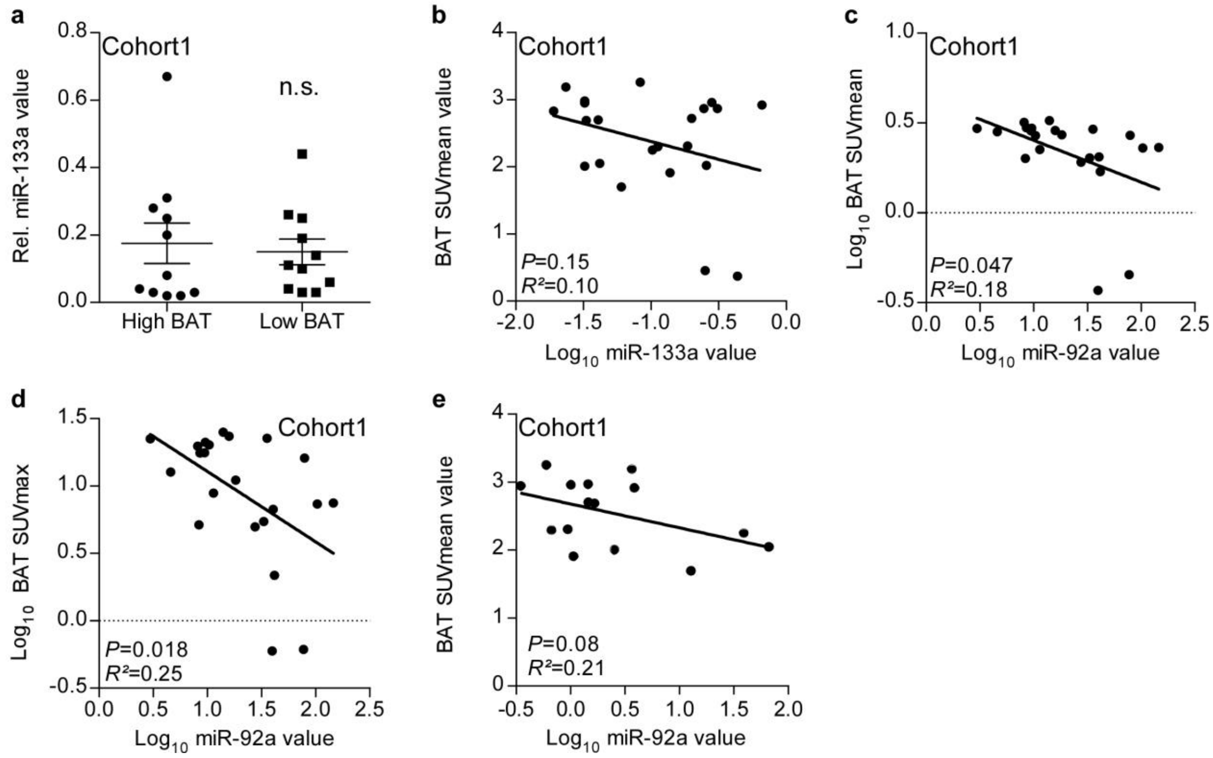

Supplementary Figure 3 Detailed analysis of miR-133a and miR-92a in exosomes from human serum, related to Figure 3

(**a**) miR-133a expression in human serum quantified by qPCR. Data was normalized to U6, and is presented as mean ± s.e.m. (unpaired, two-tailed *t*-test, n.s., not significant, n=11 per group). (**b**) Log_{10} miR-133a value is not related to BAT SUVmean value (Pearson's correlation, R^2=0.10, P=0.15, n=22). (**c**) Correlation of Log_{10} transformed BAT SUVmean and Log_{10} miR-92a (Pearson's correlation, R^2=0.18, P=0.047, n=22); related to Fig. 3c. (**d**) Log_{10} BAT SUVmax and Log_{10} miR-92a correlation (Pearson's correlation, R^2=0.25, P=0.018, n=22); related to Fig. 3d. (**e**) Correlation of BAT SUVmean and Log_{10} miR-92a of acutely cold exposed subjects (Pearson's correlation, R^2=0.21, P=0.08, n=15)

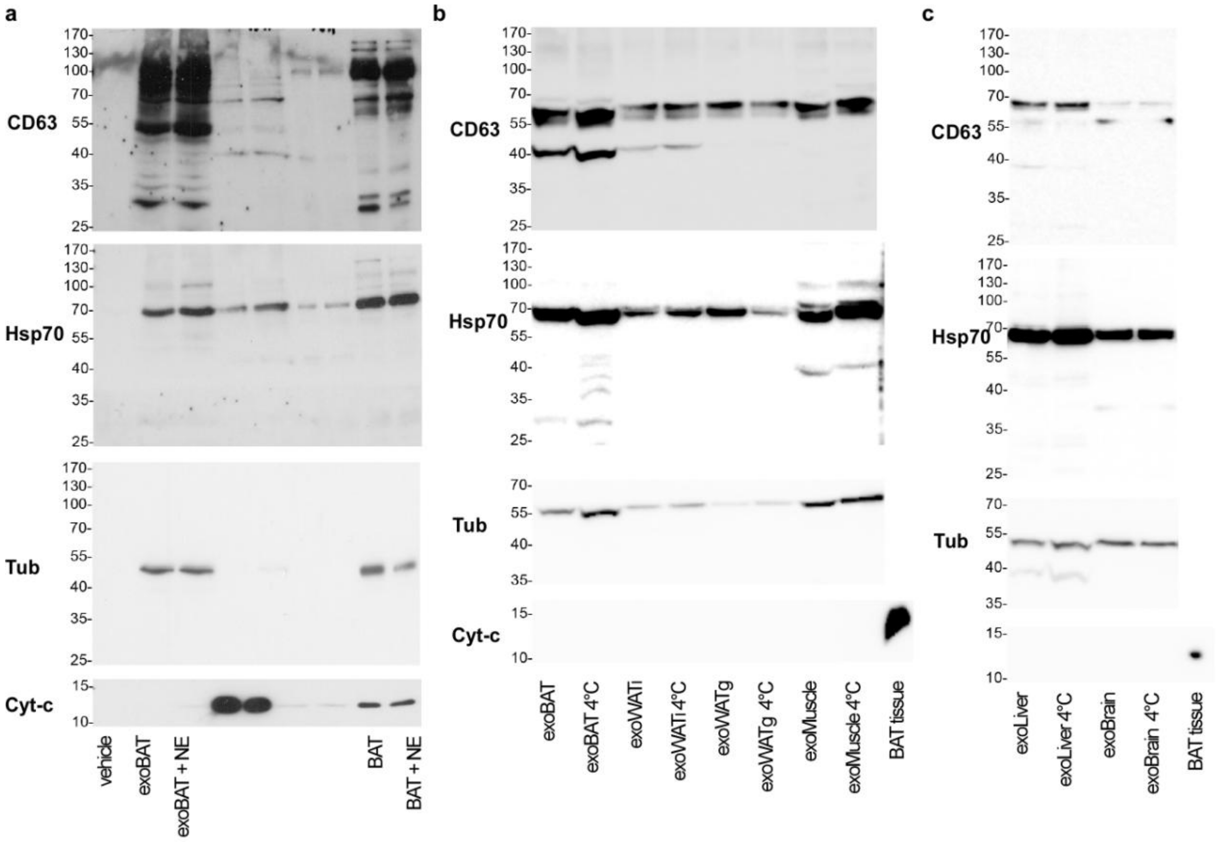

Supplementary Figure 4 Full display of Western blots, related to Figure 1 and Supplementary Figure 1

(a) Complete immunoblots of CD63, Hsp70, Tubulin (Tub) and Cytochrom-C (Cyt-c) utilized in Fig. 1b. Complete immunoblots of CD63, Hsp70, Tubulin (Tub) and Cytochrom-C (Cyt-c) utilized in Supplementary Fig. 1b (b) and (c).

miRNA candidates	cold	CL	cAMP
mmu-miR-27a-000408	65.93	1.00	
hsa-miR-149-002255	35.87	1.00	4.97
hsa-miR-93#-002139	31.30	0.67	3.94
mmu-miR-494-002365	20.20	1.00	1.75
mmu-miR-881-002609	19.25	11.18	0.11
rno-miR-146B-002755	16.93	1.56	
mmu-miR-31-000185	16.63	1.00	
mmu-miR-142-3p-000464	15.19	46.02	
mmu-miR-876-3p-002464	14.32	1.00	
mmu-miR-199a-3p-002304	12.98	0.25	
rno-miR-450a-001345	11.95	1.00	
mmu-miR-204-000508	11.64	1.00	
mmu-miR-203-000507	10.44	0.89	0.05
mmu-miR-200b-002251	10.17	1.00	
mmu-let-7e-002406	9.44	35.19	
mmu-miR-670-002020	8.89	3.04	4.96
mmu-miR-21-000397	6.32	1.00	
mmu-miR-200c-002300	6.05	1.00	0.55
mmu-miR-20a-000580	5.08	3.09	
mmu-let-7g-002282	4.92	1.38	
mmu-miR-34c#-002584	4.42	40.10	8.72
mmu-miR-145-002278	4.28	1.91	
mmu-miR-30d-000420	4.18	1.44	
mmu-miR-296-5p-000527	4.12	1.00	
rno-miR-743b-002068	3.98	6.14	1.45
mmu-miR-2146-241082_mat	3.72	2.64	
hsa-miR-493-3p-001282	3.18	2.38	
rno-miR-347-001334	3.12	0.38	
mmu-miR-26a-000405	3.09	1.68	
mmu-miR-125b-3p-002378	3.08	1.42	
mmu-miR-30a-000417	2.87	9.35	
mmu-miR-210-000512	2.75	1.65	0.13
mmu-miR-193b-002467	2.60	0.37	1.91
mmu-miR-195-000494	2.55	9.64	1.98
mmu-miR-432-241135_mat	2.40	1.16	
mmu-miR-106b-000442	2.37	2.59	
mmu-miR-26b-000407	2.36	2.02	
mmu-miR-186-002285	2.34	0.40	
hsa-miR-340-000550	2.18	1.00	
mmu-miR-1896-121128_mat	2.10	1.67	
mmu-miR-425-001516	2.05	11.13	
hsa-miR-421-002700	1.86	1.59	
mmu-miR-720-001629	1.80	1.37	

mmu-miR-19b-000396	1.78	1.21	
mmu-miR-687-001674	1.75	1.00	
mmu-miR-30e-002223	1.70	1.34	80.42
mmu-miR-741-002457	1.67	1.00	
mmu-miR-125a-5p-002198	1.61	1.40	
mmu-miR-652-002352	1.49	1.00	
mmu-miR-1937c-241011_mat	1.46	1.15	
mmu-miR-30b-000602	1.44	0.83	
mmu-miR-685-001670	1.42	1.05	2.09
mmu-miR-1942-121136_mat	1.37	1.14	
mmu-miR-1188-002866	1.36	1.76	
mmu-miR-871-002354	1.35	1.00	
mmu-miR-484-001821	1.35	1.17	1.03
mmu-miR-1894-3p-241002_mat	1.34	1.38	
mmu-miR-1937b-241023_mat	1.34	1.08	
rno-miR-7#-001338	1.32	1.00	
mmu-miR-1897-5p-121199_mat	1.30	0.61	
mmu-miR-673-001954	1.28	1.42	
mmu-miR-222-002276	1.24	0.77	27.87
rno-miR-25#-002080	1.23	1.00	
mmu-miR-532-5p-001518	1.19	1.00	
mmu-miR-615-5p-002353	1.18	1.00	
mmu-miR-501-001356	1.18	1.00	
hsa-miR-423-3P-002626	1.14	1.94	
mmu-miR-463-002662	1.10	1.38	
mmu-miR-1191-002892	1.08	2.66	
mmu-miR-486-001278	1.06	0.70	
mmu-miR-1904-121162_mat	1.03	1.69	
mmu-miR-191-002299	1.03	0.88	3.10
mmu-miR-30c-000419	1.02	0.14	
mmu-miR-151-3p-001190	1.00	10.71	
mmu-miR-339-3p-002533	1.00	10.42	27.63
mmu-miR-669m-121190_mat	1.00	9.07	
mmu-miR-101b-002531	1.00	5.32	1.82
mmu-miR-302a-000529	1.00	5.28	
mmu-miR-532-3p-002355	1.00	4.71	
mmu-miR-1971-121161_mat	1.00	4.22	
mmu-miR-297a#-002454	1.00	2.79	0.28
mmu-miR-322-001059	1.00	2.13	
mmu-miR-883b-3p-002565	1.00	2.02	
mmu-miR-376c-002450	1.00	1.85	
mmu-miR-466k-240990_mat	1.00	1.56	
rno-miR-742-002055	1.00	1.22	
mmu-miR-331-5p-002233	1.00	1.15	
mmu-miR-697-001631	1.00	1.14	2.89
hsa-miR-144-002676	1.00	1.04	

U6 snRNA-001973	0.99	1.09	0.99
mmu-miR-140-001187	0.98	0.98	
rno-miR-196c-002049	0.97	0.97	
hsa-miR-223-000526	0.96	0.92	
rno-miR-632-241110_mat	0.95	0.98	
mmu-miR-16-000391	0.93	0.76	1.42
rno-miR-489-001353	0.92	1.22	0.74
mmu-miR-2134-241120_mat	0.92	0.65	
mmu-miR-667-001949	0.91	1.92	0.29
Mamm U6-001973	0.90	1.09	
mmu-miR-342-3p-002260	0.88	1.96	2.93
hsa-miR-412-001023	0.87	2.63	
mmu-miR-374-5p-001319	0.87	2.43	
mmu-miR-409-3p-002332	0.84	0.40	0.33
rno-miR-327-001328	0.84	1.30	
mmu-miR-1951-121165_mat	0.83	1.41	
mmu-miR-96-000186	0.82	0.82	
mmu-miR-139-5p-002289	0.82	0.39	
mmu-miR-543-001298	0.81	0.81	
mmu-miR-17-002308	0.81	0.47	
mmu-miR-467b-001671	0.81	0.81	
mmu-miR-146a-000468	0.81	0.81	3.85
mmu-miR-488-001659	0.81	0.66	
mmu-miR-1905-121196_mat	0.80	0.83	
mmu-miR-196a#-002477	0.80	1.09	0.50
mmu-miR-141#-002513	0.79	0.79	0.75
mmu-miR-1982.1-121157_mat	0.78	0.78	
mmu-miR-1956-121129_mat	0.77	0.77	
mmu-miR-24-000402	0.77	0.34	0.93
mmu-miR-1969-121131_mat	0.76	0.96	
mmu-miR-320-002277	0.74	0.90	4.38
mmu-miR-872-002264	0.73	1.06	
rno-miR-673-002054	0.73	0.73	
mmu-miR-877#-002548	0.72	0.95	2.37
hsa-miR-200c-000505	0.72	0.03	
rno-miR-190b-002048	0.68	0.78	
mmu-miR-223-002295	0.67	0.88	
mmu-miR-301a-000528	0.67	0.08	
mmu-miR-152-000475	0.66	0.70	
mmu-miR-465C-5P-002654	0.65	0.53	
mmu-miR-24-2#-002494	0.65	0.65	75.38
mmu-miR-467b-001684	0.64	0.64	
mmu-miR-92a-000430	0.64	0.43	0.01
hsa-miR-140-3p-002234	0.63	0.72	
mmu-miR-155-002571	0.63	0.72	
mmu-miR-467F-002886	0.61	21.32	

mmu-miR-188-3p-002106	0.61	0.61	
rno-miR-664-001323	0.59	0.10	8.44
mmu-miR-574-3p-002349	0.58	0.04	44.03
mmu-miR-1961-197391_mat	0.57	0.57	
mmu-miR-1274a-121150_mat	0.57	0.70	
mmu-miR-106a-002459	0.56	0.84	
hsa-miR-378-000567	0.55	0.62	1.64
mmu-miR-181A-2#-002687	0.55	0.55	
ath-miR159a-000338	0.55	0.55	
mmu-miR-29a-002112	0.53	1.70	
mmu-miR-150-000473	0.51	0.69	
mmu-miR-25-000403	0.50	1.29	
mmu-miR-451-001141	0.49	0.81	
mmu-miR-470#-002589	0.49	0.92	
mmu-miR-2138-241080_mat	0.49	0.15	
mmu-miR-29c-000587	0.45	0.70	
mmu-miR-1901-121183_mat	0.43	0.49	
mmu-miR-328-000543	0.43	0.48	2.36
hsa-miR-143-000466	0.41	0.89	
mmu-miR-466b-3-3p-002500	0.41	0.91	
mmu-miR-1982.2-121154_mat	0.39	2.17	
mmu-miR-133a-002246	0.38	0.44	0.03
mmu-miR-467a-001826	0.38	2.13	
mmu-miR-146b-001097	0.35	0.42	
mmu-miR-19a-000395	0.33	0.31	
mmu-miR-126-3p-002228	0.33	0.40	
mmu-miR-743a-002469	0.29	0.29	
mmu-miR-29b-000413	0.27	0.27	
rno-miR-1-002064	0.25	0.19	2.44
mmu-miR-126-5p-000451	0.25	1.62	
mmu-miR-692-001679	0.18	1.00	
mmu-miR-429-001077	0.17	0.17	
mmu-miR-1939-121180_mat	0.15	0.45	
mmu-miR-450b-5p-001962	0.14	0.56	
mmu-miR-133b-002247	0.13	0.13	
mmu-miR-93-001090	0.12	0.01	
mmu-miR-465a-5p-001082	0.12	0.22	
mmu-miR-197-000497	0.10	0.10	
mmu-miR-504-002084	0.09	0.04	
mmu-miR-215-001200	0.09	0.52	
mmu-miR-125b-5p-000449	0.09	0.05	
rno-miR-878-002070	0.08	0.08	
mmu-let-7d-002283	0.06	0.06	
mmu-miR-218-000521	0.05	0.05	
hsa-miR-214-000517	0.05	0.05	11.40
mmu-miR-188-5p-002320	0.05	0.15	1.18

mmu-miR-331-3p-000545	0.05	0.05	
mmu-miR-130a-000454	0.04	0.04	
hsa-miR-30e-3p-000422	0.04	0.04	
mmu-miR-1-002222	0.03	0.16	
hsa-miR-30a-3p-000416	0.03	0.03	48.46
mmu-miR-2182-241119_mat	0.02	0.18	
mmu-miR-326-001061	0.01	0.51	0.88

Supplementary Table 1 Changes in miRNAs patterns in exosomes from mice or BA after activation, related to Figure 2

Serum exosomal miRNAs up- or down-regulated more than 2-fold in either CL316,243-treated (CL) or in cold-exposed mice (cold) (versus wild type controls) and exosomal miRNAs up- or down-regulated more than in cAMP-treated BA (versus untreated BA controls). miRNAs up-regulated are indicated in red; down-regulated in blue; undetected miRNAs are listed in black. Investigated candidates are highlighted in white.

	Serum-exo<br>*in vivo*		BA-exo<br>*in vitro*
	CL-316.243	Cold	cAMP
miR-34c*	40.101	4.421	8.722
miR-339-3p	10.419	1.000	27.631
miR-149	1.000	35.871	4.970
miR-93*	0.667	31.295	3.940
miR-409-3p	0.396	0.844	0.326
miR-92a	0.427	0.637	0.014
miR-133a	0.441	0.383	0.032

Supplementary Table 2 Expression of the seven deregulated miRNA candidates in mice, related to Figure 2

Seven miRNA candidates found in serum of mice with active BAT (CL-316.243 or cold) and in brown adipocytes treated with cAMP respective to wild type mice and untreated brown adipocytes. Given are Fold Changes (FC) of Relative Quantitation (RQ) compared to wild type mice and untreated brown adipocytes.

ID	AGE	BMI	SEX	Fat	Glucose Uptake Rate	BAT SUVmax	Pre-BSm	Post-BSm	ΔBsm	Predicted ΔBsm	Pre-LgmiR92	Post-LgmiR92	ΔLgmiR92	Acute cold sample
2	20	19.90	0	15.80	0.00	0.60	0.37	1.96	1.59	0.45	1.60	1.08	-0.51	
4	25	23.40	1	12.60	10.22	22.63	2.92	2.53	-0.38	-0.11	1.55	1.68	0.13	x
5	22	20.30	0	14.80	5.40	8.86	2.25	2.01	-0.24	0.24	1.05	0.78	-0.27	x
6	24	21.70	0	25.70	4.92	20.19	2.69	3.11	0.42	-0.22	1.02	1.27	0.25	x
7	20	21.90	0	22.10	1.93	4.97	1.91	2.75	0.84	0.47	1.44	0.91	-0.53	x
8	19	19.90	1	12.50	10.44	17.68	2.87	2.95	0.09	0.03	0.97	0.94	-0.03	
10	23	20.80	0	12.80	5.67	21.04	2.96	3.85	0.89	-0.01	0.98	1.00	0.02	x
11	21	18.40	1	13.40	10.67	22.37	2.95	2.55	-0.39	-0.02	0.47	0.49	0.02	x
13	25	25.70	1	12.50	5.46	5.45	2.02	2.46	0.44	0.38	1.52	1.09	-0.43	
14	18	20.40	1	8.40	11.00	23.40	2.87	3.46	0.59	0.17	1.20	1.00	-0.20	

Supplementary Table 3 Cohort 1 subject characteristics for participants in the 10 day cold acclimation study, related to Figure 3

Shown are age, BMI in kg m^{-2}, sex (0, female; 1, male), Fat in kg, Glucose Uptake Rate in µmol $100g^{-1}$ min^{-1}, BAT SUVmax, BAT SUVmean before (Pre-BSm) and after cold acclimation (Post-BSm), delta BAT SUVmean (ΔBsm), the predicted BAT SUVmean (Predicted ΔBsm; Fig. 3d), Log_{10} miR-92a (Pre-LgmiR92 or Post-LgmiR92) and $ΔLog_{10}$ miR-92a, respectively. Additionally it is listed whether an acute cold sample was taken utilized for Supplemental Fig. 3e.

ID	AGE	BMI	SEX	Fat	GlucoseUptakeRate	BAT SUVmean	BAT SUVmax	Log_{10}miR-92a	Acute cold sample
1	19	23.82	0	22.28	7.09	2.83	12.70	4.57	
2	20	21.76	1	7.54	12.23	3.26	25.04	13.96	x
3	21	21.89	1	10.71	7.56	2.72	11.03	18.25	
4	20	21.43	1	11.46	11.01	3.19	19.76	8.11	x
5	23	20.85	0	15.74	6.61	2.70	16.09	78.84	x
6	20	18.54	1	6.09	9.57	2.98	17.59	8.55	x
7	23	19.62	0	19.93	3.01	2.01	5.16	8.35	x
8	21	20.18	0	15.33	0.00	0.00	0.61	77.04	
9	19	22.29	0	17.94	4.43	2.05	6.71	40.39	x
10	24	21.20	1	13.23	0.00	1.70	2.16	41.51	x
11	26	21.25	1	10.98	5.32	2.31	7.47	145.28	x
12	19	22.30	0	19.04	3.76	2.30	7.33	103.01	x

Supplemementary Table 4 Cohort 1 subject characteristics for 12 lean participants in the thermoneutral study, related to Figure 3

Shown are age, BMI in kg m^{-2}, sex (0, female; 1, male), Fat in kg, Glucose Uptake Rate in µmol $100g^{-1}$ min^{-1}, BAT SUVmean, BAT SUVmax, Log_{10} miR-92a. Additionally it is listed whether an acute cold sample was taken utilized for Supplemental Fig. 3e

ID	AGE	BMI	SEX	Fat	GlucoseUptakeRate	Log_{10}miR-92a
1	47	24.7	0	35.1	17.1	1.67
2	30	26.6	1	22.6	1	1.93
3	46	24.5	0	34.6	2.2	2.20
4	21	18.9	0	22.1	10.7	1.93
5	38	20.5	0	28	4.9	2.28
6	40	24.2	0	32.9	3.9	1.49
7	48	20.4	1	19.3	0.9	2.11
8	50	22.7	0	33.3	1.4	2.24
9	51	23.5	0	34.5	3.7	1.84
10	44	23.6	0	33	6.3	2.22
11	28	24.2	0	-	32.7	1.28
12	35	-	1	-	8.7	1.56
13	25	23	1	16.6	7.9	1.49
14	25	24.7	0	25.8	21	1.22
15	24	20.3	0	25.8	22.8	1.80
16	31	25.1	0	36.1	26.3	1.11
17	25	25.1	1	20.1	9.5	2.53
18	39	28.9	1	18.6	23.1	1.60
19	43	23.4	0	34	8.4	2.60

Supplementary Table 5 Cohort 2 subject characteristics for 19 lean participants in the second thermoneutral study, related to Figure 3

Shown are age, BMI in kg m^{-2}, sex (0, female; 1, male), Fat in kg, Glucose Uptake Rate in µmol $100g^{-1}$ min^{-1}, and Log_{10} miR-92a.

Correlation Cohort1										
		AGE	BMI	Sex	Fat	GlucoseUptakeRate	BAT SUVmean	BAT SUVmax	Log_{10} miR-92a	Log_{10} miR-133a
AGE	Pearson	1	.247	.139	.026	-.220	-.056	-.110	.292	.159
	t-test (2-tail)		.268	.538	.909	.326	.806	.626	.188	.479
	N	22	22	22	22	22	22	22	22	22
BMI	Pearson	.247	1	.164	.235	-.041	.065	-.118	.270	.157
	t-test (2-tail)	.268		.465	.294	.856	.773	.600	.224	.485
	N	22	22	22	22	22	22	22	22	22
Sex	Pearson	.139	.164	1	-.749**	.509*	.369	.294	.035	.259
	t-test (2-tail)	.538	.465		.000	.016	.091	.184	.876	.245
	N	22	22	22	22	22	22	22	22	22
Fat	Pearson	.026	.235	-.749**	1	-.536*	-.292	-.353	.012	-.306
	t-test (2-tail)	.909	.294	.000		.010	.187	.107	.959	.166
	N	22	22	22	22	22	22	22	22	22
GlucoseUptakeRate	Pearson	-.220	-.041	.509*	-.536*	1	.863**	.883**	-.505*	-.112
	t-test (2-tail)	.326	.856	.016	.010		.000	.000	.016	.618
	N	22	22	22	22	22	22	22	22	22
BAT SUVmean	Pearson	-.056	.065	.369	-.292	.863**	1	.858**	-.510*	-.315
	t-test (2-tail)	.806	.773	.091	.187	.000		.000	.015	.153
	N	22	22	22	22	22	22	22	22	22
BAT SUVmax	Pearson	-.110	-.118	.294	-.353	.883**	.858**	1	-.528*	-.114
	t-test (2-tail)	.626	.600	.184	.107	.000	.000		.012	.613
	N	22	22	22	22	22	22	22	22	22
Log_{10} miR-92a	Pearson	.292	.270	.035	.012	-.505*	-.510*	-.528*	1	.432*
	t-test (2-tail)	.188	.224	.876	.959	.016	.015	.012		.045
	N	22	22	22	22	22	22	22	22	22
Log_{10} miR-133a	Pearson	.159	.157	.259	-.306	-.112	-.315	-.114	.432*	1
	t-test (2-tail)	.479	.485	.245	.166	.618	.153	.613	.045	
	N	22	22	22	22	22	22	22	22	22

Supplementary Table 6 Pearson Correlation for 22 lean participants of the thermoneutral study, related to Figure 3

Shown are age, BMI in kg m^{-2}, sex (0, female; 1, male), Fat in kg, Glucose Uptake Rate in µmol $100g^{-1}$ min^{-1}, BAT SUVmean, BAT SUVmax, Log_{10} miR-92a and the respective Log_{10} miR-133a. Values are Pearson correlation coefficients (*P**≤0.05, ***P*≤0.01).

Correlation Cohort2							
		AGE	BMI	Sex	Fat	GlucoseUptakeRate	Log_{10} miR-92a
AGE	Pearson	1	.089	-.186	.560*	-.479*	.354
	t-test (2-tail)		.727	.447	.019	.038	.137
	N	19	18	19	17	19	19
BMI	Pearson	.089	1	.324	.042	.242	-.271
	t-test (2-tail)	.727		.190	.873	.333	.276
	N	18	18	18	17	18	18
Sex	Pearson	-.186	.324	1	-.814**	-.192	.036
	t-test (2-tail)	.447	.190		.000	.430	.883
	N	19	18	19	17	19	19
Fat	Pearson	.560*	.042	-.814**	1	-.040	.026
	t-test (2-tail)	.019	.873	.000		.880	.921
	N	17	17	17	17	17	17
GlucoseUptakeRate	Pearson	-.479*	.242	-.192	-.040	1	-.630**
	t-test (2-tail)	.038	.333	.430	.880		.004
	N	19	18	19	17	19	19
Log_{10} miR-92a	Pearson	.354	-.271	.036	.026	-.630**	1
	t-test (2-tail)	.137	.276	.883	.921	.004	
	N	19	18	19	17	19	19

Supplementary Table 7 Pearson Correlation for 19 lean participants of a second thermoneutral study, related to Figure 3

Pearson Correlation for 19 lean participants of a second thermoneutral study. Shown are age, BMI in kg m^{-2}, sex (0, female; 1, male), Fat in kg, C-reactive Protein in mgL^{-1}, Glucose Uptake Rate in µmol $100g^{-1}$ min^{-1} and Log_{10} miR-92a. Values are Pearson correlation coefficients ($P*\leq 0.05$, $**P\leq 0.01$).
